# Data on inflammasome gene polymorphisms of patients with sporadic malignant melanoma in a Brazilian cohort

**DOI:** 10.1016/j.dib.2016.11.053

**Published:** 2016-11-24

**Authors:** Wanessa Cardoso da Silva, Telma M. Oshiro, Daniel Coelho de Sá, Dilcilea D.G.S. Franco, Cyro Festa Neto, Alessandra Pontillo

**Affiliations:** a Laboratory of Medical Investigation in Dermatology and Immunodeficiences - LIM 56, Department of Dermatology, Faculty of Medicine, University of Sao Paulo, Avenida Dr. Eneas de Carvalho Aguiar, 470 – Prédio 2 - 3° andar – CEP:05403-000 - Cerqueira César, Sao Paulo, Brazil; b Department of Dermatology, Faculty of Medicine, University of Sao Paulo, Avenida Dr. Eneas de Carvalho Aguiar, 500 – CEP: 05403-000 Cerqueira Cesar, Sao Paulo, Brazil; c Laboratory of Immunogenetics, Department of Immunology, Institute of Biomedical Sciences, University of Sao Paulo, Avenida Prof. Lineu Prestes, 1730 – 05508-000 Cidade Universitaria, Sao Paulo, Brazil

## Abstract

This article presents data related to our another article entitled, **Genotyping and differential expression analysis of inflammasome genes in sporadic malignant melanoma reveal novel contribution of CARD8, IL1B and IL18 in melanoma susceptibility and progression (**W.C. Silva, T.M. Oshiro, D.C. Sá, D.D.G.S. Franco, C. Festa Neto, A. Pontillo**,** 2016) [2]. Data presented here refers to the distribution of selected inflammasome SNPs in a Brazilian case/control cohort. We have identified 4 inflammasome related Single Nucleotide Polymorphisms (SNPs) for *CARD8* (rs6509365); *IL1B* (rs1143643) and *IL18* (rs5744256 and rs1834481) related to melanoma susceptibility/protection. This data can serve as a potential prognostic marker in sporadic malignant melanoma.

**Specifications Table**

**Table:** 

Subject area	*Genetics*
More specific subject area	*Immunogenetics*
Type of data	*Tables, figures*
How data was acquired	*ABI Prism 7300 Real Time PCR equipment (Applied Biosystems, Thermoscientific, USA), SDS 2.3 software (Applied Biosystems, Thermoscientific, USA), R software*(*www.r-project.org*), *Haploview software*
Data format	*Raw, analyzed*
Experimental factors	*Determination of clinical data of patients, extraction of DNA from buffy coat and genetic polymorphism parameters*
Experimental features	*Analysis of polymorphism in inflammasome genes in sporadic malignant melanoma patients and healthy controls*
Data source location	*Sao Paulo, Brazil*
Data accessibility	*The data is available with this article*

**Value of the data**

• Presence or absence of a particular polymorphism in inflammasome genes can drive an individual´s susceptibility to melanoma.
• This dataset provides some selected inflammasome related SNPs’ frequencies in a Brazilian case/control melanoma cohort and its association with clinical outcomes.
• Comparison of this dataset with other cohort dataset can help to elucidate the contribution of inflammasome genes in the development of and progression to sporadic malignant melanoma.

## 1 Data

A Brazilian case/control SMM cohort was studied concerning frequencies of selected inflammasome SNPs in *NLRP1*, *NLRP3*, *CARD8*, *IL1B* and *IL18* genes and minor allele frequencies (MAF) with respective Hardy–Weinberg *p*-values were calculated.

Case/control analysis were performed and distribution of alleles for each selected SNP, as well as Odd Ratios (OR), haplotypes*, Linkage disequilibrium* analysis were determined.

**Fig. 1:**
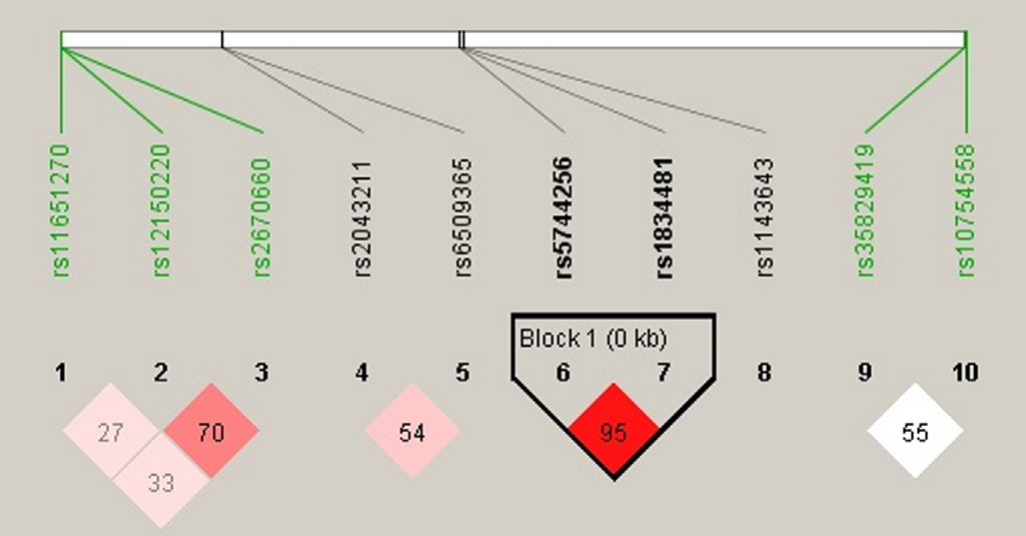
Linkage disequilibrium results for single-nucleotide polymorphisms examined in case/control study. Haploview plot showed D’/LOD values.

Patients were stratified according to histological tumor type, invasiveness and skin type were represented (Fig. 1).

## 2 Experimental design, materials and methods

Refer to the associated article [2] for detailed methods (Table 1, Table 2, Table 3, Table 4, Table 5).

## Figures and Tables

**Table 1:** **SNPs frequencies in case/control cohort.** Minor allele frequencies (MAF) with respective Hardy–Weinberg *p*-values (HW p) for studied SNPs are reported in case (SMM) and controls (HC). Hapmap project MAF (or 1000 Genome MAF where indicated) for Caucasian and African population are included. MAF for SNPs studied by Verma et al., 2012^a^[1] are also included with respective *p*-value for comparisons with SNPs frequencies in studied Brazilian cohort^a^.

**Gene**	**SNP ID**	**Allele**	**SMM**	**HW p**	**HC**	**HW p**	**CEU**	**YRI**	**SMM** (Verma et al. [1])	**HC** (Verma et al. [1])
*NLRP1*	rs12150220	T	0.44	0.071	0.46	0.079	0.46	0.02	0.52 (0.020)	0.20 (<2exp-16)
	rs2670660	G	0.51	0.180	0.46	0.485	0.35^⁎^	0.32^⁎^		
	rs11651270	C	0.50	0.059	0.41	0.216	0.47	0.50		
*NLRP3*	rs35829419	A	0.04	1.0	0.04	1.0	0.06	na	0.08 (4.2exp-8)	0.06 (1.0exp-6)
	rs10754558	G	0.41	0.878	0.38	0.070	0.36	0.23		
*CARD8*	rs2043211	T	0.29	0.203	0.38	0.855	0.27	0.16	0.34 (0.524)	0.36 (0.026)
	rs6509365	G	0.25	0.157	0.40	0.373	0.28	0.32		
*IL1B*	rs1143643	T	0.40	1.0	0.37	0.804	0.39	0.15		
*IL18*	rs5744256	G	0.18	0.803	0.20	0.177	0.22	na		
	rs1834481	G	0.17	0.628	0.20	0.103	0.23	na		

a data from 1000 Genome.

**Table 2:** **Association results for inflammasome polymorphisms in sporadic malignant melanoma.** Genotype frequencies are reported as well as unadjusted *p*-values (*p*), *p*-values adjusted for age, sex and ethnicity (*p*_adj_) and respective Odds Ratio (OR) and 95% confidence intervals (95% CI). Statistically significant results (*p*<0.005) are indicated in bold characters. SMM: sporadic malignant melanoma; HC: healthy controls; Ref: reference genotype.

**Gene**	**SNP ID**	**Genotypes**	**SMM (*n*=198)**	**HC (*n*=142)**	***p***	**OR (95%IC)**	***p***_**adj**_	**OR (95%IC)**

*NLRP1*	rs12150220	T/T	0.23	0.25	0.887	0.87 (0.48–1.58)	0.673	0.86 (0.43–1.69)
		A/T	0.42	0.42		0.98 (0.58–1.64)		1.15 (0.64–2.08)
		A/A	0.35	0.33		Ref		Ref
	rs2670660	G/G	0.28	0.19	0.145	1.54 (0.81–2.93)	0.166	1.69 (0.81–3.54)
		A/G	0.45	0.53		0.87 (0.51–1.49)		0.91 (0.49–1.69)
		A/A	0.27	0.28		Ref		Ref
	rs11651270	C/C	0.28	0.19	0.155	1.84 (0.98–3.44)	0.132	1.97 (0.97–3.99)
		T/C	0.43	0.43		1.25 (0.73–2.12)		1.11 (0.61–2.01)
		T/T	0.29	0.37		Ref		Ref
*NLRP3*	rs35829419	A/A	0	0	0.911	–	0.810	–
		C/A	0.07	0.07		1.05 (0.44 -2.54)		1.13 (0.42–3.02)
		C/C	0.93	0.93		Ref		Ref
	rs10754558	G/G	0.17	0.18	0.310	1.14 (0.60–2.16)	0.467	1.20 (0.58–2.46)
		C/G	0.48	0.40		1.46 (0.89- 2.40)		1.42 (0.81–2.48)
		C/C	0.35	0.42		Ref		Ref
***CARD8***	rs2043211	T/T	0.06	0.13	0.043	0.37 (0.16–0.83)	0.102	0.39 (0.15–0.98)
		A/T	0.45	0.49		0.73 (0.46–1.18)		0.69 (0.40–1.19)
		A/A	0.49	0.38		Ref		Ref
	***rs6509365***	*G/G*	*0.08*	*0.18*	***3.1 exp-4***	*0.28 (0.13–0.58)*	***1.7 exp-4***	*0.35 (0.15–0.80)*
		*A/G*	*0.33*	*0.44*		*0.48 (0.29–0.78)*		*0.41 (0.24–0.72)*
		*A/A*	*0.59*	*0.38*		*Ref*		*Ref*
*IL1B*	rs1143643	T/T	0.16	0.12	0.700	1.45 (0.59–3.56)	0.581	1.76 (0.56–5.53)
		C/T	0.48	0.49		1.06 (0.58–1.95)		1.02 (0.46–2.27)
		C/C	0.36	0.39		Ref		Ref
*IL18*	rs5744256	G/G	0.03	0.06	0.589	0.58 (0.19–1.72)	0.235	0.39 (0.12–1.29)
		A/G	0.29	0.28		1.03 (0.62 -1.70)		0.77 (0.44–1.34)
		A/A	0.67	0.66		Ref		Ref
	rs1834481	G/G	0.05	0.07	0.755	0.64 (0.17 -2.36)	0.244	0.40 (0.09–1.86)
		C/G	0.23	0.25		0.87 (0.43–1.74)		0.58 (0.27–1.28)
		C/C	0.73	0.69		Ref		Ref

**Table 3:** **Association results for*****IL18*****rs5744256-rs1834481 haplotypes.** Frequencies for SMM patients (case) and healthy donors (control) as well as *p*-values are reported for resulted rs5744256-rs1834481 haplotypes. Only haplotypes with frequency >0.05 are included in GLM analysis. SMM: sporadic malignant melanoma; HC: healthy controls.

Haplotypes Rs5744256-rs1834481	Case/Control Frequencies	*p*	*p*_adj_

C-A	0.79/0.80	ref	ref
G-G	0.17/0.19	0.8647	0.8512
rare	0.04/0.01	0.1663	0.1381

**Table 4:** **Association results for inflammasome polymorphisms in melanoma patients stratified for histological tumor type**. Gene symbol, polymorphism identification number (SNP ID), *p*-values adjusted for sex, age and ethnicity are reported for three comparisons: superficial spreading melanoma (SSM) versus nodular melanoma (NM), SSM versus lentigo malignant melanoma (LMM) and SSM versus melanoma “*in situ”.* Statistically significant values are indicated in bold characters. nd: not determined.

**Gene**	**SNP ID**	**SSM (*n*=77)/ NM (*n*=30)**	**SSM (*n*=77)/LMM (*n*=21)**	**SSM (*n*=77)/*****in situ*****(*n*=29)**
		Dominant model of inheritance	Recessive model of inheritance	Recessive model of inheritance
*NLRP1*	rs12150220	**0.018**	0.109	0.639
	rs2670660	0.229	0.475	0.820
	rs11651270	**0.003**	0.060	0.08
*NLRP3*	rs35829419	nd	nd	nd
	rs10754558	0.303	**0.020**	0.952
*CARD8*	rs2043211	**0.005**	0.108	0.792
	rs6509365	**0.040**	0.983	0.600
*IL1B*	rs1143643	0.815	0.904	**0.020**
*IL18*	rs5744256	0.903	0.937	**0.050**
	rs1834481	0.176	0.946	**0.030**

**Table 5:** **Association results for inflammasome polymorphisms in melanoma patients according to invasiveness and skin type.** Gene symbol, polymorphism identification number (SNP ID), multivariate analysis *p*-values adjusted for sex, age at diagnosis and ethnicity are reported for SMM patients according to invasiveness (Breslow index), with less invasive (<2 mm) or more invasive (≥2 mm) tumors; or with sun-sensitive or less sun-sensitive skin types. Statistically significant values are indicated in bold characters. nd: not determined.

**Gene**	**SNP ID**	**Tumor invasiveness**	**Skin type**
		**≥ 2 mm (*n*=30)/<2 mm(*n*=133)**	**Sun-sensitive (*n*=94)/Less sun-sensitive (*n*=57)**
*NLRP1*	rs12150220	0.690	0.880
	rs2670660	0.645	0.950
	rs11651270	0.616	0.836
			
*NLRP3*	rs35829419	nd	nd
	rs10754558	0.537	0.986
			
*CARD8*	rs2043211	0.256	0.692
	rs6509365	0.409	0.624
*IL1B*	rs1143643	**0.004**	0.978
			
*IL18*	rs5744256	0.970	**0.003**
	rs1834481	0.455	**0.027**

## References

[1] Verma D., Bivik C., Farahani E., Synnerstad I., Fredrikson M., Enerback C., Rosdahl I., Soderkvist P. (2012). Inflammasome polymorphisms confer susceptibility to sporadic malignant melanoma. Pigment Cell Melanoma Res..

[2] W. Silva, T. Oshiro, D. Sá, F.DDGS, C. Festa Netoand A. PontilloGenotyping and differential expression analysis of inflammasome genes in sporadic malignant melanoma reveal novel contribution of *CARD8, IL1B* and *IL18* in melanoma susceptibility and progression. Cancer Genetics. Submitted 10.1016/j.cancergen.2016.09.004 27810076

